# Phylogenetic and Molecular Epidemiological Studies Reveal Evidence of Multiple Past Recombination Events between Infectious Laryngotracheitis Viruses

**DOI:** 10.1371/journal.pone.0055121

**Published:** 2013-02-01

**Authors:** Sang-Won Lee, Joanne M. Devlin, John F. Markham, Amir H. Noormohammadi, Glenn F. Browning, Nino P. Ficorilli, Carol A. Hartley, Philip F. Markham

**Affiliations:** 1 Asia-Pacific Centre for Animal Health, Faculty of Veterinary Science, The University of Melbourne, Parkville, Victoria, Australia; 2 Asia-Pacific Centre for Animal Health, Faculty of Veterinary Science, The University of Melbourne, Werribee, Victoria, Australia; 3 National ICT Australia (NICTA) Victoria Research Laboratory, Department of Electrical and Electronic Engineering, School of Engineering, The University of Melbourne, Victoria, Australia; British Columbia Centre for Excellence in HIV/AIDS, Canada

## Abstract

In contrast to the RNA viruses, the genome of large DNA viruses such as herpesviruses have been considered to be relatively stable. Intra-specific recombination has been proposed as an important, but underestimated, driving force in herpesvirus evolution. Recently, two distinct field strains of infectious laryngotracheitis virus (ILTV) have been shown to have arisen from independent recombination events between different commercial ILTV vaccines. In this study we sequenced the genomes of additional ILTV strains and also utilized other recently updated complete genome sequences of ILTV to confirm the existence of a number of ILTV recombinants in nature. Multiple recombination events were detected in the unique long and repeat regions of the genome, but not in the unique short region. Most recombinants contained a pair of crossover points between two distinct lineages of ILTV, corresponding to the European origin and the Australian origin vaccine strains of ILTV. These results suggest that there are two distinct genotypic lineages of ILTV and that these commonly recombine in the field.

## Introduction

Herpesviruses are highly host-specific double stranded DNA viruses that have been isolated from most animal species, including mammals, birds, reptiles and fish. The *Herpesviridae* have genomes ranging in size from 124 to 241 kbp [1] and been classified into three subfamilies, the *Alpha-, Beta-* and *Gammaherpesvirinae*. Classically, sequences obtained from a single gene or multiple concatenated genes have been used for phylogenetic analyses of herpesviruses. More recently developments in high-throughput sequencing techniques have resulted in the availability of complete genome sequences for comparative complete genomic analyses of herpesviruses [2], [3], [4].

Infectious laryngotracheitis virus (ILTV, gallid herpesvirus 1) belongs to the subfamily *Alphaherpesvirinae* (genus *Iltovirus*) and causes acute upper respiratory tract disease in chickens [5]. Phylogenetic analysis, incorporating a time scale, of amino acid sequences of eight genes from herpesviruses has suggested that the *Iltovirus* genus (including psittacid herpesvirus 1 (PsHV1) and ILTV) separated from the mammalian alphaherpesviruses around 200 million years (Mys) ago, in agreement with the theory that herpesviruses have co-evolved with their hosts [1]. Comparative analysis of the complete genome sequences of ILTV and PsHV1 has confirmed that they are closely related and distinct within the *Alphaherpesvirinae* [6]. Recently, the complete genome sequences of a number of ILTV strains have been determined using high-throughput sequencing techniques and these data have been used to perform phylogenetic and comparative genomic studies [2], [7], [8], [9].

In Australia three commercial live ILTV vaccines (the Australian origin SA2 and A20 vaccines and the European origin Serva vaccine) are registered for use. The SA2 vaccine strain (Pfizer, Australia) is an attenuated ILTV field strain isolated from South Australia in 1950 [10], [11], [12]. The A20 vaccine strain (Pfizer, Australia) was produced by serial passages of the SA2 ILTV strain in primary chick embryo cell cultures in order to decrease its residual virulence [13]. The Serva vaccine strain was registered in Australia in 2006 [14].

Australian ILTV field strains and vaccine strains have been classified into nine genotypes (classes 1 to 9) using polymerase chain reaction and restriction fragment length polymorphism (PCR-RFLP) analysis [15], [16]. The Australian and European origin ILTV vaccines have been assigned into classes 1 and 7, respectively [15], [16]. Class 2 viruses, represented by the V1-99 strain, which was isolated from a hen in a commercial laying flock in 1999 [15], were the predominant isolates obtained from disease outbreaks in Australia until 2008 [16]. Strain CSW-1 (class 4), which was originally isolated in 1970 from layer birds [17], is currently used as a standard laboratory strain [18], [19].

In a previous report we analysed and compared the complete genome sequences of two Australian origin ILTV vaccines and a European origin ILTV vaccine strain. The genome sequences of the two Australian origin vaccines differed by only 23 nucleotide substitutions, even though one of the strains was derived from the other after more than twenty-five serial passages in cell culture and embryonated eggs. Although similar to each other, these two Australian vaccine strains showed considerable divergence (0.8% nucleotide difference) from the genomic sequence of the European origin ILTV vaccine strain. This is higher than the 0.1–0.5% nucleotide difference reported between the most distantly related strains of varicella zoster viruses (VZV), which is a human alphaherpesvirus with the same genomic sequence arrangement as ILTV [20]. It has been hypothesised that viral evolution in a geographically isolated environment could explain the high levels of divergence between the Australian and European origin strains of ILTV [2]. However, the absence of wild native red jungle fowl, which is the major ancestor of domesticated chickens, on the continent of Australia [21], [22] and the relatively short history of the Australian poultry industry (chickens were first introduced in the late 18^th^ century) [23] makes it unlikely that this divergence could have occurred in such a short period of time.

The aim of this study was to better understand the molecular epidemiology of ILTV by analysing a broader range of ILTV sequence data, including currently available complete or partial genomic sequences of ILTV. The complete genome sequences of two Australian ILTV field strains (V1-99 and CSW-1) were determined using high-throughput sequencing and included in these analyses. Additionally, we aimed to identify the origins of the parent strain of the Australian ILTV vaccines by searching for similar genotypes among publically available nucleotide sequences from ILTV strains isolated in other countries.

## Results

### Complete Genome Sequences of the Australian ILTV Field Strains V1-99 and CSW-1

**Figure 1:**
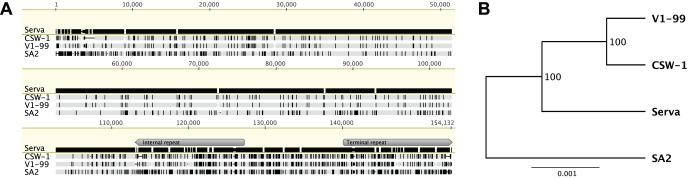
Nucleotide sequence alignment and phylogenetic tree analysis for the complete genomes of ILTV strains. (A) Alignment of the complete genome sequences of Australian ILTV field strains and vaccines was performed using MAFFT. The Serva ILTV sequence was set as the reference sequence. Vertical lines indicate SNPs compared to the Serva sequence and dashes indicate sequence gaps. (B) Phylogenetic tree generated using the alignment and the UPGMA method. One hundred bootstrap replicates were used to assess the significance of the tree topology. A bar indicates nucleotide substitutions per site.

Sequencing data from the V1-99 and CSW-1 strains of ILTV obtained by high-throughput sequencing were assembled *de novo* and mapped to the complete genome sequence of the Serva vaccine strain. Sequence gaps were filled and ambiguous sequences clarified using Sanger sequencing. The size of the ILTV genomes ranges from 150,335 to 153,952 bp (for the unique long region, 109,575–113,174 bp; for the unique short region, 13,094–13,099 bp; and for the internal and terminal repeat regions, 12,283–14,547 bp). The size of the strain V1-99 genome was 153,631 bp, similar to that of the Serva strain (153,645 bp). The V1-99 and Serva strains shared 99.6% nucleotide sequence identity. The CSW-1 strain genome was somewhat smaller (151,671 bp) due to three large deletions within the unique long region (1,227 bp) and both the internal and terminal repeat regions (463 bp) (Fig. 1A). Excluding the genomic regions containing the deletions, the genome of the CSW-1 strain had 99.8% nucleotide sequence identity with that of V1-99. The G+C content of the CSW-1 and V1-99 ILTV was 48.1%. The genomes of both strains had 79 predicted ORFs. Complete genome alignment and phylogenetic analysis of the V1-99 and CSW-1 strains revealed a close genetic relationship between them. Interestingly, these two Australian field strains were more closely related to the European origin vaccine strain (Serva ILTV) than the Australian origin vaccine strain (SA2 ILTV) as shown in the phylogenetic tree inferred using the Unweighted Pair Group Method with Arithmetic Mean (UPGMA) algorithm (Fig. 1B).

### Alignment and Phylogenetic Analysis of Nucleotide and Amino Acid Sequences of Predicted ORFs

**Figure 2:**
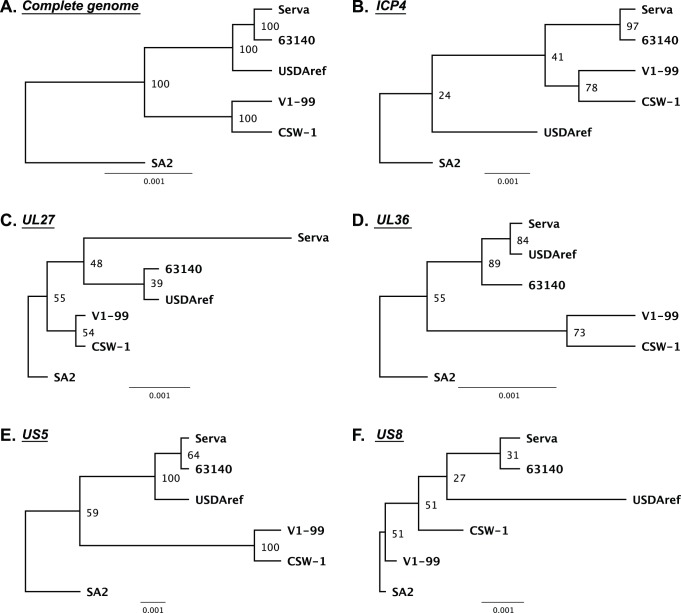
Phylogenetic trees showing the relationships between ILTV strains from Australia and overseas, based on analysis of specific ORFs. The rooted trees were generated from alignments of complete genome sequences (A), or the complete amino acid sequences of the ICP4 (B), UL27 (C), UL36 (D), US5 (E) and US8 (F) genes, using the UPGMA method, with SA2 ILTV as the root. One hundred bootstrap replicates were used to assess the significance of the tree topology. A bar indicates the sequence substitutions per site.

In order to further understand the relationship between ILTV vaccine strains and Australian field strains of ILTV, complete genome sequence alignments were performed and phylogenetic trees were inferred using the six complete genomic sequences of ILTV currently available. This analysis included a European origin vaccine (Serva), an Australian origin vaccine (SA2), two Australian field strains (V1-99 and CSW-1) and two strains from the USA (63140 and USDAref). The sequences were examined in order to identify relevant genes that could be used to differentiate between all six strains (Fig. 2).

All predicted ORFs were extracted from the complete genome alignments and separate phylogenetic trees were inferred from the nucleotide sequences of each ORF. Similarly, alignments of amino acid sequences for all the predicted ORFs were performed separately and phylogenetic trees were inferred. The UL21, 32, 34 and 43 genes of all six ILTV strains were found to share 100% nucleotide and amino acid sequence identity. The UL3, 4, 13, 14, 18, 24, 31, 33, 40 and 49 genes of the six strains had differing nucleotide sequences, but were predicted to have 100% amino acid sequence identity. The ICP4, UL27, UL36, US5 and US8 genes showed the most variability within the six strains of ILTV and the six strains could be distinguished using the nucleotide and/or amino acid sequences of these genes (Fig. 2). The UL27 and the ICP4 genes were selected to perform further analysis, as a considerable number of nucleotide sequences for these two genes were available in the NCBI database.

**Figure 3:**
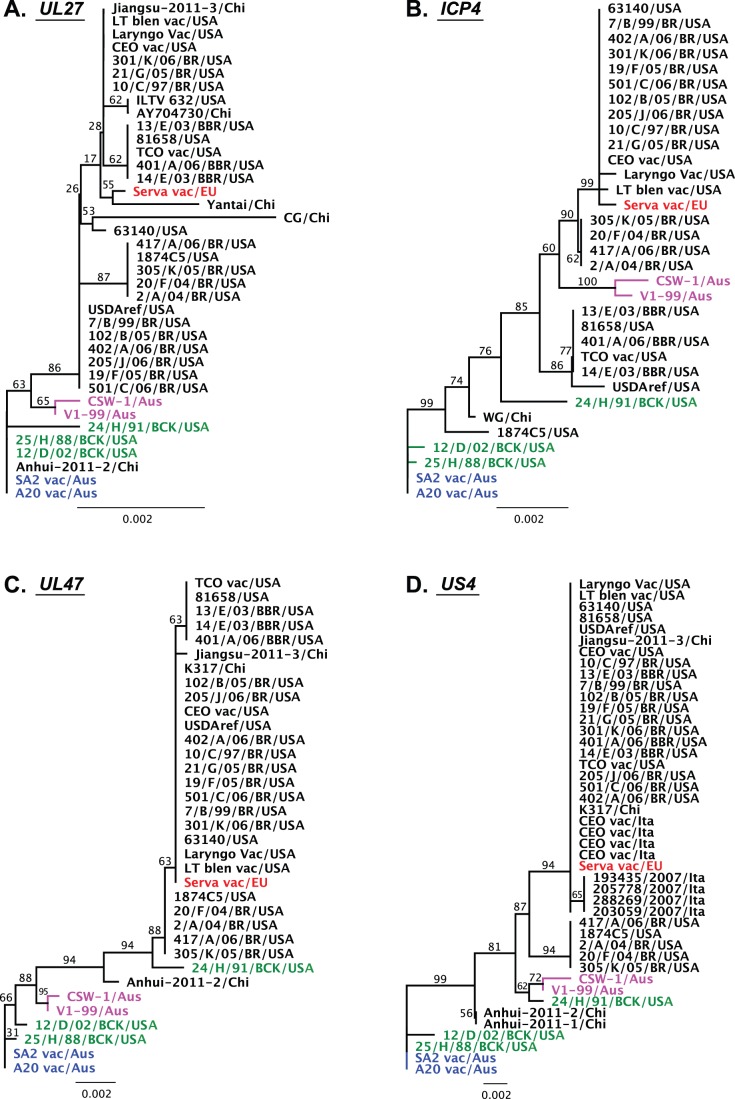
Phylogenetic analyses of specific genes of ILTV. Maximum likelihood phylogenetic trees were generated using alignments of nucleotide sequences of the UL27 (A), ICP4 (B), UL47 (C) and US4 (D) genes obtained from the NCBI sequence database. One hundred bootstrap replicates were used to assess the significance of the tree topologies and the number of replicate trees containing each specific branch in the consensus tree is shown next to that branch. Pink text indicates Australian field isolates and green text indicates USA backyard flock strains of ILTV. The Serva vaccine strain is indicated in red text and the Australian origin vaccine SA2 and A20 strains in blue text. vac, vaccine; EU, Europe; USA, United States of America; Aus, Australia; Chi, China; Ita, Italy.

All available nucleotide sequences for the ICP4 and UL27 genes of ILTV from the NCBI database were aligned and phylogenetic analyses were performed (Fig. 3A and B). The European origin vaccine grouped closely with the USA vaccines, and with the USA field isolates. The Australian field strains of ILTV clustered separately, but they had inconsistent relationships with the European and Australian origin vaccines in these phylogenetic analyses. The Australian field strains clustered closely with the Australian origin vaccine in the UL27 tree (Fig. 3A), while they were closely related to the European origin vaccine in the ICP4 tree (Fig. 3B). Interestingly, the Australian origin vaccine strains clustered as an outgroup together with USA field strains originally isolated from backyard flocks.

**Figure 4:**
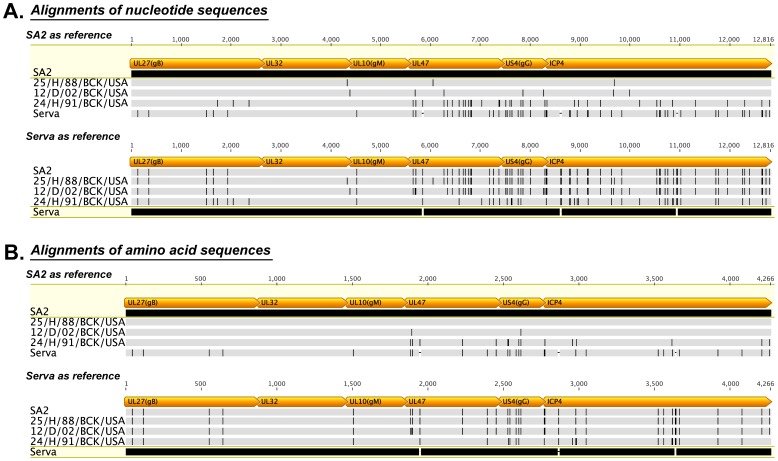
Alignments of concatenated nucleotide and amino acid sequences from USA backyard flock strains and vaccine strains of ILTV. All available nucleotide (A) and amino acid (B) sequences from USA backyard flock strains of ILTV were obtained from the NCBI database and concatenated following the order of the genes in the ILTV genome. The locations of the ORFs are indicated. The concatenated sequences were compared with those of the European (Serva) and Australian (SA2) origin vaccines. The sequences of the SA2 or Serva strains were set as the reference. A vertical line indicates sequence differences from the reference sequences. Dashes indicate sequence gaps.

To confirm the close relationship between the Australian origin vaccine strain and strains isolated from backyard flocks in the USA, concatenated nucleotide and amino acid sequences were generated using all available sequences for each of these strains. This included the complete sequences of the UL27, UL32 and UL10 genes from the unique long region, the UL47 and US4 genes from the unique short region, and the complete sequence of the ICP4 gene from the repeat region. Surprisingly, one of the strains isolated from backyard flocks in the USA (25/H/88/BCK) shared 100% amino acid sequence identity (with three synonymous single nucleotide polymorphisms, SNPs) with the concatenated sequence of the Australian origin vaccine. Another strain isolated from backyard flocks in the USA (12/D/02/BCK) differed in only two amino acid residues from the Australian origin vaccine strain (Fig. 4). These results suggest that the parent strain of the Australian origin ILTV vaccine strains may have been introduced into Australia from the USA. The lack of genetic divergence between the strains indicates that this may have occurred relatively recently, though this needs further investigation.

### Evidence of Historical Recombination between ILTV Strains

A possible mosaic pattern of relationship between the genome sequences of the Australian field strains of ILTV was detected in the phylogenetic analyses of the UL27 and ICP4 genes (Fig. 3A and B). In addition, the sequence of one of the ILTV strains from backyard flocks in the USA (24/H/91/BCK) also showed a mosaic pattern of relationship with other strains, especially within the UL47 gene, the sequence of which was closely related to that of the Serva strain (Fig. 4). This suggested it might be a historical recombinant between a Serva-like strain and a USA backyard flock strain.

**Figure 5:**
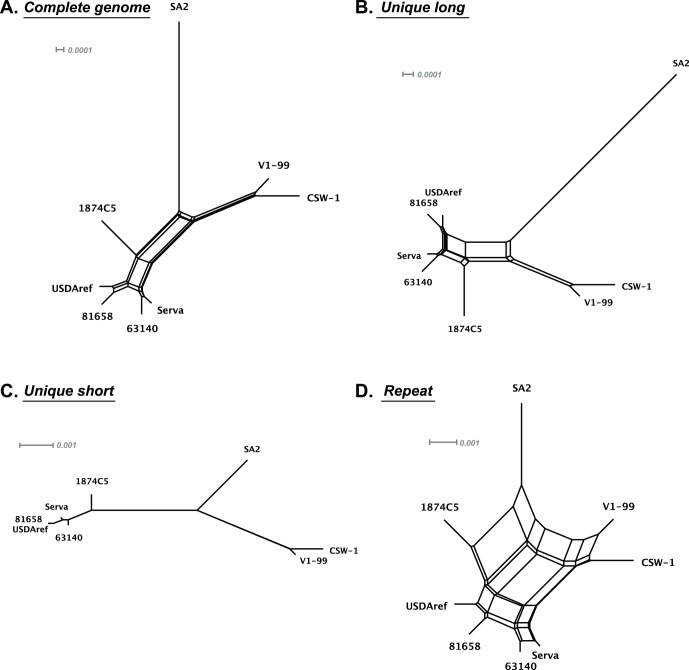
ILTV recombination networks. Phylogenetic networks were generated from nucleotide alignments of the complete genome sequences (A), the unique long region (B), the unique short region (C), and the repeat region (D) using SplitsTree. The multiple reticulate networks indicate historical recombination events between the different strains of ILTV. A bar indicates the sequence substitutions per site. The Phi test for detecting recombination, as implemented in SplitsTree, was also performed on all regions. This was highly significant for A, B and D regions (P values<0.001).

**Figure 6:**
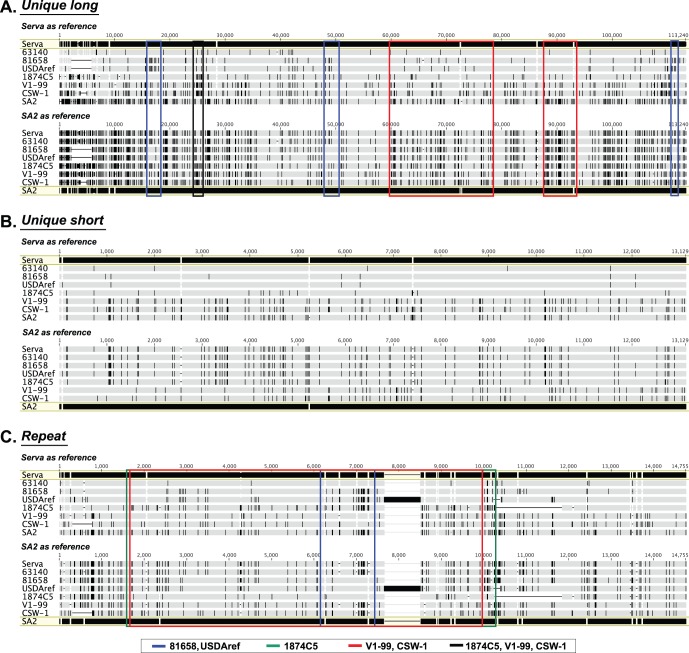
Nucleotide sequence alignments for three genomic regions of ILTV strains and suspected recombination regions. Nucleotide sequence alignments of the unique long region (A), the unique short region (B), and the repeat region (C) of eight ILTV strains were performed using MAFFT. The sequences of the Serva or SA2 strains were set as the reference. A vertical line indicates single nucleotide differences from the reference sequences. Dashes indicate sequence gaps. The genomic regions suspected as being recombinations are marked on the alignments with colour-coded boxes. The genomic region of high identity between the Australian field strains and the 1874C5 is indicated with a black box.

To confirm the mosaic patterns of these relationships and detect the suspected recombination events responsible, we performed additional phylogenetic analyses on the nucleotide sequences of the UL47 and US4 genes (Fig. 3C and D). As expected, the 24/H/91/BCK ILTV strain grouped with the European origin Serva strain in the UL47 and US4 trees, but grouped with the Australian origin SA2 vaccine strain and other USA backyard flock isolates in both the UL27 and the ICP4 phylogenetic trees. Similarly, the Australian field strains (V1-99 and CSW-1) grouped with the European origin Serva strain in the ICP4 and US4 trees, but grouped with the Australian origin SA2 strain in the UL27 and the UL47 trees (Fig. 3). As strains very similar or identical to both Serva and SA2 were found among USA strains, we hypothesised that there was also a possibility of natural recombination occurring between ILTV strains in the USA, similar to that recently recognized in Australia [24]. Recombination networks were generated using eight complete genome sequences (excluding the terminal repeat region) to assess the likelihood that recombination events had occurred (Fig. 5). Surprisingly, these networks showed evidence of significant recombination events (P<0.0001), or very close relationships, between all field strains of ILTV, including those from both the USA and Australia, and commercial vaccines. Evidence of recombination was detected in the unique long and repeat regions, but not in the unique short region. Recombination appeared to be more frequent in the repeat region (Fig. 5B-D). Variable genetic polymorphisms were identified in the alignments of the unique long and repeat regions, while all strains of ILTV had a distinct genetic profile in the unique short region. Excluding the 63140 strain, which has a very high sequence similarity to the Serva strain, all other field strains of ILTV appeared to have mosaic genomes with major genomic regions with high similarity to one reference strain and small genomic regions with much greater similarity to a different reference strain (Fig. 6).

**Figure 7:**
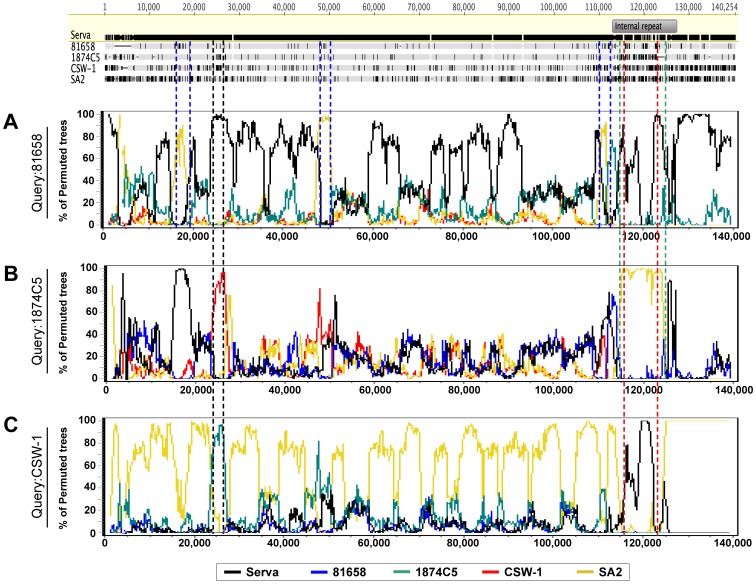
Bootscan analyses for ILTV recombination crossover points. Alignments and bootscan analyses were produced using five complete genome sequences of different strains of ILTV. The bootscan analyses were performed using Simplot, with the query sequences 81685 (A), 1874C5 (B) and CSW-1 (C). The crossover points detected are marked on the alignments of the complete genome sequences with color-coded dashed lines. The black dashed lines indicate regions of high identity in 1874C5 and CSW-1.

In order to define recombination crossover points, Bootscan analyses were performed using complete genome alignments of five ILTV strains (Serva, SA2, CSW-1, 1874C5 and 81658). Bootscan analyses of the 1874C5 and CSW-1 strains revealed pairs of crossover points in the repeat region, while the 81658 strain had multiple tightly grouped pairs of crossover points in the unique long region (Fig. 7). Most of the genomic regions that were suggested as recombination regions in the complete genome alignment (Fig. 6) were confirmed by the bootscan analyses. In addition, the Australian field strains V1-99 and CSW-1 shared a distinct segment of high sequence similarity in the unique long region with the 1874C5 strain (Fig. 6 and 7).

### Estimation of Time Points of Divergence between ILTV Strains

**Figure 8:**
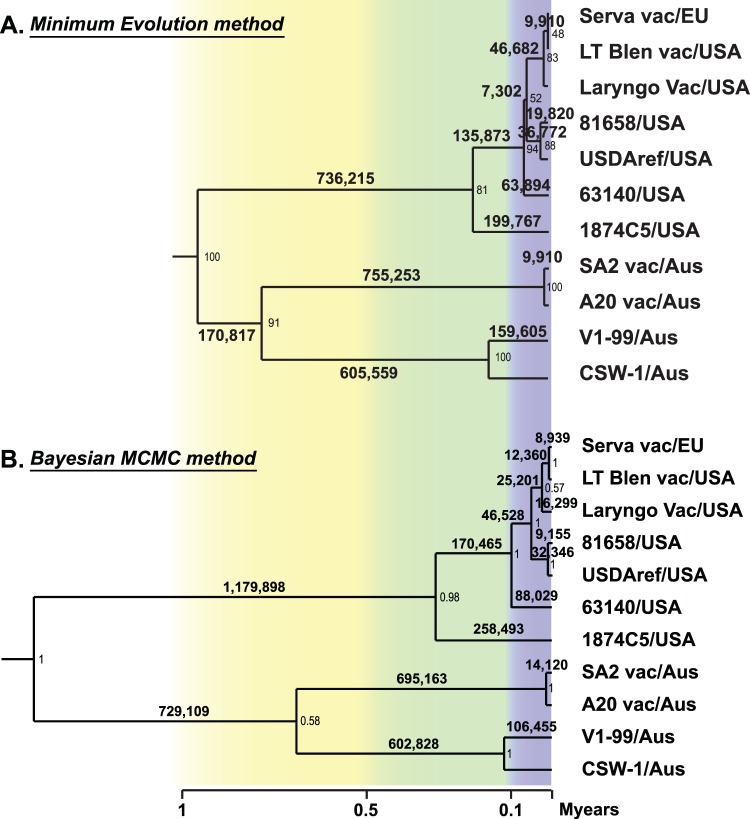
Evolutionary relationships between ILTV strains. The evolutionary history of ILTV was inferred using the Minimum Evolution method (A) and the Bayesian MCMC method (B). The number of bootstrap replicates represented by each branch of the consensus tree or the posterior probability is indicated next to each branch. The tree is drawn to scale, with branch lengths in the same units as those used to infer the tree. The estimated evolutionary times (Mys) are also indicated on the branches.

A history of recombination was not detected in the sequences located in the unique short region of the genome of the eight strains of ILTV for which complete genomic sequence was available, so the sequences of this region were used to investigate the evolutionary history of ILTV. In the resulting trees, all Australian strains of ILTV clustered together and separately from European and USA strains of ILTV (Fig. 8). The time point of divergence between these two major lineages of ILTV (European/USA and Australian), using two independent methods, was estimated to be about 0.94–1.44 Mys ago (Fig. 8). Divergence between the Australian origin vaccines and field strains was estimated to have occurred between 0.71–0.77 Mys ago. The USA genotype group VI strain (1874C5/USA) appeared to have been the first virus to diverge from the European/USA lineage and this divergence was estimated to have occurred between 0.20–0.26 Mys ago.

## Discussion

Genome wide comparative sequence analyses of closely related strains of ILTV, or of strains from specific geographical regions, have been reported previously [2], [7], [9]. However, these studies have been focused on detecting genetic determinants of pathogenicity by comparing the complete sequences of strains known to display different levels of virulence *in vivo*. In this study, we sought to perform broader phylogenetic analyses of ILTV in order to understand the evolution and molecular epidemiology of the virus. We found that the parent strains of Australian origin vaccine and Australian field strains may have been imported into Australia from the USA. Moreover, with the exception of field isolates derived from bird-to-bird passage of commercial vaccines, most field strains of ILTV isolated in Australia and in other geographic areas of the world show evidence of historical recombination events between two distinct viral lineages, represented by the Serva and SA2 strains.

The Australian origin SA2 vaccine was produced using an Australian field isolate that induced comparatively smaller pocks on the chorioallantoic membrane of embryonated hen eggs [10]. This vaccine has been used only in Australia. The geographically restricted use of this vaccine strain led to the hypothesis that the considerable divergence observed in the genomic sequence of this strain, when compared to that of the European origin Serva vaccine, resulted from geographic isolation and evolution in an isolated environment [2]. However, the results from our current study suggest an alternative hypothesis. The ILTV genome appears to be very stable, as has been described for other herpesviruses [25], [26]. The estimated evolutionary timeline we have constructed for ILTV suggests that the Serva and SA2 strains diverged well before the introduction of poultry into Australia. Thus it is likely that the divergence and independent evolution of these strains occurred elsewhere.

In order to search for an SA2-like strain of ILTV in other geographical areas, we compared the phylogenetic tree inferred from alignments of complete genome sequences with those inferred from alignments for all individual predicted ORFs. The ICP4, UL27, UL36, US5 and US8 genes were found to be sufficiently divergent to allow differentiation between distinct strains of ILTV (Fig. 2). Comparative analyses of the UL27 and ICP4 gene sequences suggested that ILTV strains isolated from backyard flocks in the USA had similar genomic sequences to those of the Australian origin SA2 vaccine strain (Fig. 3). These results suggest that ILTV strains isolated from backyard flocks in the USA and the Australian origin SA2 strain shared a common ancestor, and that this ancestor may have originated on the American continent and then been subsequently introduced into Australia.

The European origin Serva vaccine strain was recently registered in Australia and rapidly became widely used by the Australian poultry industry within a relatively short timeframe [16]. After introduction of this vaccine, two new and genotypically distinct virulent field strains were isolated from outbreaks of disease in commercial flocks and have recently been shown to be independent recombinants between Australian and European origin vaccines. Crossover points for the distinct recombination events that resulted in these viruses were detected in the unique long region of the genomes [24]. Surprisingly, more recombinants, including two historical Australian field strains and three USA field strains, were detected in the current study using the eight complete genome sequences of ILTV available in the NCBI database (Fig. 5, 6, 7). The Australian field strains V1-99 and CSW-1 were shown to have a close relationship with each other, and with the Australian origin SA2 vaccine strain in their unique long and unique short regions, but had a closer relationship with the European origin Serva vaccine strain in their repeat region sequences (Fig. 5, 6, 7), even though there are no reports of isolation or detection of Serva-like ILTV strains in Australia prior to the introduction of a vaccine based on this strain to the continent in 2007. In addition, the time point of divergence between V1-99 and CSW-1 was estimated to be around 0.11–0.16 Mys ago (Fig. 8). These results suggest that both these strains (V1-99 and CSW-1) evolved elsewhere, with a history that included recombination with a Serva-like strain, and were then independently introduced into Australia.

The Australian field strains V1-99 and CSW-1 and the USA field strain 874C5 shared a distinct sequence segment in the unique long region. This suggests that the Australian field strains and the 1874C5 strain may have shared the same (or very similar) parent strain(s) before recombination, or that these strains may have obtained this distinctive sequence segment from some other strain in independent recombination events.

Evidence of historical intra-specific recombination in alphaherpesviruses has been described previously [20], [27], [28], [29]. DNA recombination has been proposed to occur during replication of the genome of herpesviruses, a process that is still poorly understood [30]. In our current study we have shown that ILTV has a relatively low frequency of historical recombination, similar to that described for Varicella-Zoster virus (VZV), which has a lower frequency of recombination than HSV-1 [20]. This similarity between ILTV and VZV may be explained by their similar genomic architecture. Both ILTV and VZV have only one pair of repeat sequences in their genome. In contrast, HSV-1 has a genome containing two pairs of repeat sequences [31].

With the exception of the USDAref and 81658 ILTV strains, which each contained three recombination crossover points, all other ILTV recombinants contained a single pair of recombination crossover points between the Serva-like and the SA2-like parental strains, as detected by bootscan analyses. Recombination networks further supported these findings, confirming that recombination events occurred in the unique long and the repeat regions, but not in the unique short region (Fig. 5 and 7). These results indicate that there are at least two distinct conserved genotypes of ILTV, and that these genotypes can recombine with each other. It is possible that other distinct ILTV genotypes may still remain undetected. In the current study, we included specific gene sequences obtained from Asian isolates, but complete genome sequences for Asian ILTV isolates have not yet been determined. As domesticated chickens originated from wild chickens in Southeast Asia [21], complete genome sequences of ILTVs isolated from wild chickens in the same geographical area may be useful in gaining a better understanding of the evolution and epidemiology of ILTV.

In conclusion, the origins of Australian strains of ILTV have been determined using complete genomic sequence analyses of multiple strains of ILTV and wider phylogenetic analyses. Moreover, evidence for significant recombination events in most field strains of ILTV was identified by recombination network and bootscan analyses. We found that the Serva-like and SA2-like lineages of ILTV have recombined frequently over the history of ILTV evolution. This, taken together with data from our previous studies [24], highlights the risk of new distinct ILTV strains arising in chickens infected simultaneously with different genotypes of ILTV.

## Materials and Methods

### Viruses

Pock purified V1-99 (six passages from isolation) and a laboratory stock of CSW-1 ILTV (approximately twenty passages from isolation) [32] were propagated by allantoic sac inoculation of specific-pathogen-free embryonated hen eggs. Viruses were purified from the allantoic fluid as previously described [8].

### High-throughput Sequencing and Genome Assembly

Total viral genomic DNA preparation, high-throughput sequencing and genome assembly were performed as previously described [2]. The software package Geneious [33] was used to manually curate the alignments of the *de novo* assembled contigs and to produce a consensus sequence using the Serva ILTV genomic sequence (Genbank accession HQ_630064) as reference. Any sequence gaps or ambiguous regions of sequence were confirmed by Sanger sequencing using BDT version 3.1 (Applied Biosystems). The complete genome sequences of the V1-99 and CSW-1 strains of ILTV have been deposited in the NCBI GenBank database under the accession numbers JX646898 and JX646899.

### Sequences and Alignment

Complete genome sequences, nucleotide sequences and amino acid sequences used in this study are summarized in Table S1. Complete genome sequences of the V1-99 and CSW-1 strains of ILTV were determined as part of this study. The complete genome sequences of nine other ILTV strains, as well as the nucleotide and amino acid sequences of individual ILTV genes, including those of the UL27, UL47, US4 and ICP4 genes, from viruses isolated in different geographic areas were obtained from the NCBI database. The NCBI reference genome sequence of ILTV (NC_006623) was excluded in the present analysis, as it has been assembled from partial sequences obtained from six different strains of ILTV and does not represent a complete single ILTV genome sequence for comparison. Complete genome sequence alignments were performed using Multiple Alignment with Fast Fourier Transformation (MAFFT) v6 [34]. Shorter nucleotide sequence alignments and amino acid sequence alignments were performed using ClustalW v2 [35]. Phylogenetic analyses on the complete genome sequence alignments were performed using the UPGMA algorithm in Geneious with the Tamura-Nei nucleotide substitution model [36]. Phylogenetic analyses on the complete amino acid sequence alignments of the ICP4, UL27, UL36, US5 and US8 genes were performed using the UPGMA algorithm with the Jukes-Cantor substitution model [37]. The maximum-likelihood phylogenetic trees for the nucleotide sequence alignments of the UL27, UL47, US4 and ICP4 genes were generated using PhyML version 2.4.4 [38] with the Tamura-Nei substitution model. All phylogenetic analyses were assessed statistically by analysis of one hundred bootstrap replicates.

### Recombination Analysis

To detect evidence of historical recombination events between ILTV strains, recombination networks were generated using SplitsTree 4 [39]. The nucleotide sequences of the whole genome (excluding the terminal repeat sequence), the unique long, the unique short and the internal repeat genome regions obtained from eight strains of ILTV (the Australian origin vaccine, SA2; the Australian field strains, V1-99 and CSW-1; the European origin vaccine, Serva; a genotype V USA field strain related to the chicken embryo origin vaccine strain, 63140; the USDA reference strain; a genotype III USA field strain related to the tissue culture origin vaccine, 81658; and a genotype VI USA field strain, 1874C5) were used to generate separate alignments and then the phylogenetic networks were determined using the alignments. Statistical analyses of the recombination networks were performed using the Phi test [40]. In addition, alignments of the unique long, the unique short and the internal repeat genome regions from the eight strains of ILTV were scanned manually to examine regions of most similarity between each of the genomes, to identify any mosaic relationships between the genomes. Bootscan analysis was performed to detect crossover points for recombination events using Simplot [41] with a 3000 bp window size and a 200 bp step size for the complete ILTV genomes. For the bootscan analysis only five strains of ILTV were used as representatives of each cluster (the SA2, Serva, CSW-1, 81658 and 1874C5 strains).

### Estimation of Divergence Time Points between ILTVs

To estimate the time points of divergence between ILTV strains, the Minimum Evolution [42] and Bayesian Markov chain Monte Carlo (MCMC) inference methods [43] were implemented using the nucleotide sequence alignment of all available full sequences of the unique short genomic region of ILTVs (a total of 11 strains), excluding the recent Australian recombinant viruses [24]. PsHV-1 was used as outgroup with a setting of 105 Mys as the time of divergence between ILTV and PsHV1, based on the estimated time of divergence of their respective hosts [44]. The Minimum Evolution analysis was performed in Molecular Evolutionary Genetics Analysis (MEGA) 5 [45] using 100 bootstrap replications. The tree was drawn to scale, with branch lengths using the same units of evolutionary distance (the number of base substitutions per site) as those used to infer the phylogenetic tree. The evolutionary distances were computed using the Tamura-Nei method and the rate of variation among sites was modelled using a gamma distribution (shape parameter = 4). The Bayesian MCMC analysis was performed in Bayesian Evolutionary Analysis by Sampling Trees (BEAST) v1.7.1 [46] using the general time-reversible (GTR) nucleotide substitution model and a gamma distribution (shape parameter = 4). Divergence times were estimated under a relaxed molecular clock with an uncorrelated exponential distribution of rates. The time to the most recent common ancestor (TMRCA) of ILTV and PsHV1 was set as 105 Mys. The evolutionary and coalescent parameters were estimated under the Bayesian skyline model [47]. MCMC simulations were performed with 10 million generations and subsampling every 1,000 generations. The phylogenetic tree was generated after removing a 20% burn-in.

## Supporting Information

Table S1 **ILTV genome and gene sequences used in this study.**
(DOC)
