# Supplementary material for: Phylogenetic and Molecular Epidemiological Studies Reveal Evidence of Multiple Past Recombination Events between Infectious Laryngotracheitis Viruses
Source: PLoS One. 2013 Feb 1;8(2):e55121. doi: 10.1371/journal.pone.0055121 (PMC3562231; doi:10.1371/journal.pone.0055121)
Supplement: Table S1 — ILTV genome and gene sequences used in this study. (DOC) [file pone.0055121.s001.doc]

**Table S1. ILTV genome and gene sequences used in this study**

|  | GenBank accession number, name and | Sequence | Reference |
| --- | --- | --- | --- |
|  | origin of ILTV strains^*^ | type |  |
| Complete genome | HQ630064 (Serva vac/EU) | Nucleotide | [1,2,3,4] |
| sequences | JQ083493 (LT Blen vac/USA) |  |  |
|  | JQ083494 (Laryngo Vac/USA) |  |  |
|  | JN542535 (81658/USA) |  |  |
|  | JN542534 (USDAref/USA) |  |  |
|  | JN542536 (63140/USA) |  |  |
|  | JN542533 (1874C5/USA) |  |  |
|  | JN596962 (SA2 vac/Aus) |  |  |
|  | JN596963 (A20 vac/Aus) |  |  |
|  | JX646898 (V1-99/Aus) |  |  |
|  | JX646899 (CSW-1/Aus) |  |  |
| UL27 | AY704730 (AY704730/Chi) | Nucleotide | [5,6,7] |
| (glycoprotein B) | DQ118666 (Yantai/Chi) |  |  |
|  | DQ812546 (CG/Chi) |  |  |
|  | EU104966 (TCO vac/USA) |  |  |
|  | EU104967 (13/E/03/BBR/USA) |  |  |
|  | EU104968 (14/E/03/BBR/USA) |  |  |
|  | EU104969 (401/A/06/BBR/USA) |  |  |
|  | EU104970 (10/C/97/BR/USA) |  |  |
|  | EU104971 (301/K/06/BR/USA) |  |  |
|  | EU104972 (21/G/05/BR/USA) |  |  |
|  | EU104973 (CEO vac/USA) |  |  |
|  | EU104974 (25/H/88/BCK/USA) |  |  |
|  | EU104975 (205/J/06/BR/USA) |  |  |
|  | EU104976 (501/C/06/BR/USA) |  |  |
|  | EU104977 (7/B/99/BR/USA) |  |  |
|  | EU104978 (102/B/05/BR/USA) |  |  |
|  | EU104979 (402/A/06/BR/USA) |  |  |
|  | EU104980 (19/F/05/BR/USA) |  |  |
|  | EU104981 (417/A/06/BR/USA) |  |  |
|  | EU104982 (305/K/05/BR/USA) |  |  |
|  | EU104983 (2/A/04/BR/USA) |  |  |
|  | EU104984 (20/F/04/BR/USA) |  |  |
|  | EU104985 (12/D/02/BCK/USA) |  |  |
|  | EU104986 (24/H/91/BCK/USA) |  |  |
|  | JN969089 (Anhui-2011-2/Chi) |  |  |
|  | JN969090 (Jiangsu-2011-3/Chi) |  |  |
|  | X56093 (ILTV 632/USA) |  |  |
|  | ABX59532 (12/D/02/BCK/USA) | Amino |  |
|  | ABX59533 (24/H/91/BCK/USA) | acid |  |
|  | ABX59521 (25/H/88/BCK/USA) |  |  |
| ICP4 | DQ995291 (WG/Chi) | Nucleotide | [6,7,8] |
|  | EU104899 (25/H/88/BCK/USA) |  |  |
|  | EU104900 (CEO vac/USA) |  |  |
|  | EU104901 (19/F/05/BR/USA) |  |  |
|  | EU104902 (21/G/05/BR/USA) |  |  |
|  | EU104903 (301/K/06/BR/USA) |  |  |
|  | EU104904 (10/C/97/BR/USA) |  |  |
|  | EU104905 (401/A/06/BBR/USA) |  |  |
|  | EU104906 (14/E/03/BBR/USA) |  |  |
|  | EU104907 (13/E/03/BBR/USA) |  |  |
|  | EU104908 (TCO vac/USA) |  |  |
|  | EU104910 (24/H/91/BCK/USA) |  |  |
|  | EU104911 (12/D/02/BCK/USA) |  |  |
|  | EU104912 (2/A/04/BR/USA) |  |  |
|  | EU104913 (305/K/05/BR/USA) |  |  |
|  | EU104914 (417/A/06/BR/USA) |  |  |
|  | EU104915 (20/F/04/BR/USA) |  |  |
|  | EU104916 (205/J/06/BR/USA) |  |  |
|  | EU104917 (402/A/06/BR/USA) |  |  |
|  | EU104918 (102/B/05/BR/USA) |  |  |
|  | EU104919 (7/B/99/BR/USA) |  |  |
|  | EU104920 (501/C/06/BR/USA) |  |  |
|  | ABX59446 (25/H/88/BCK/USA) | Amino |  |
|  | ABX59457 (24/H/91/BCK/USA) | acid |  |
|  | ABX59458 (12/D/02/BCK/USA) |  |  |
| UL47 | EU104855 (21/G/05/BR/USA) | Nucleotide | [6,7] |
|  | EU104856 (301/K/06/BR/USA) |  |  |
|  | EU104857 (10/C/97/BR/USA) |  |  |
|  | EU104858 (401/A/06/BBR/USA) |  |  |
|  | EU104859 (14/E/03/BBR/USA) |  |  |
|  | EU104860 (13/E/03/BBR/USA) |  |  |
|  | EU104861 (TCO vac/USA) |  |  |
|  | EU104863 (402/A/06/BR/USA) |  |  |
|  | EU104864 (7/B/99/BR/USA) |  |  |
|  | EU104865 (102/B/05/BR/USA) |  |  |
|  | EU104866 (501/C/06/BR/USA) |  |  |
|  | EU104867 (205/J/06/BR/USA) |  |  |
|  | EU104868 (19/F/05/BR/USA) |  |  |
|  | EU104869 (20/F/04/BR/USA) |  |  |
|  | EU104870 (417/A/06/BR/USA) |  |  |
|  | EU104871 (305/K/05/BR/USA) |  |  |
|  | EU104872 (2/A/04/BR/USA) |  |  |
|  | EU104873 (12/D/02/BCK/USA) |  |  |
|  | EU104874 (24/H/91/BCK/USA) |  |  |
|  | EU104875 (CEO vac/USA) |  |  |
|  | EU104876 (25/H/88/BCK/USA) |  |  |
|  | JN969105 (Anhui-2011-2/Chi) |  |  |
|  | JN969106 (Jiangsu-2011-3/Chi) |  |  |
|  | JN969107 (K317/Chi) |  |  |
|  | ABX59420 (12/D/02/BCK/USA) | Amino |  |
|  | ABX59421 (24/H/91/BCK/USA) | acid |  |
|  | ABX59423 (25/H/88/BCK/USA) |  |  |
| US4 (glycoprotein G) | EU104943 (24/H/91/BCK/USA) | Nucleotide | [6,7,9] |
|  | EU104944 (12/D/02/BCK/USA) |  |  |
|  | EU104945 (2/A/04/BR/USA) |  |  |
|  | EU104946 (305/K/05/BR/USA) |  |  |
|  | EU104947 (417/A/06/BR/USA) |  |  |
|  | EU104948 (20/F/04/BR/USA) |  |  |
|  | EU104949 (19/F/05/BR/USA) |  |  |
|  | EU104950 (402/A/06/BR/USA) |  |  |
|  | EU104951 (102/B/05/BR/USA) |  |  |
|  | EU104952 (501/C/06/BR/USA) |  |  |
|  | EU104953 (7/B/99/BR/USA) |  |  |
|  | EU104954 (205/J/06/BR/USA) |  |  |
|  | EU104955 (25/H/88/BCK/USA) |  |  |
|  | EU104956 (TCO vac/USA) |  |  |
|  | EU104957 (13/E/03/BBR/USA) |  |  |
|  | EU104958 (14/E/03/BBR/USA) |  |  |
|  | EU104960 (401/A/06/BBR/USA) |  |  |
|  | EU104961 (10/C/97/BR/USA) |  |  |
|  | EU104962 (301/K/06/BR/USA) |  |  |
|  | EU104963 (CEO vac/USA) |  |  |
|  | EU104964 (21/G/05/BR/USA) |  |  |
|  | HM230770 (193435/2007/Ita) |  |  |
|  | HM230771 (203059/2007/Ita) |  |  |
|  | HM230772 (205778/2007/Ita) |  |  |
|  | HM230773 (288269/2007/Ita) |  |  |
|  | HM230774 (CEO vac/Ita) |  |  |
|  | HM230775 (CEO vac/Ita) |  |  |
|  | HM230776 (CEO vac/Ita) |  |  |
|  | HM230777 (CEO vac/Ita) |  |  |
|  | JN969092 (Anhui-2011-1/Chi) |  |  |
|  | JN969093 (Anhui-2011-2/Chi) |  |  |
|  | JN969094 (Jiangsu-2011-3/Chi) |  |  |
|  | JN969107 (K317/Chi) |  |  |
|  | ABX59490 (24/H/91/BCK/USA) | Amino |  |
|  | ABX59491 (12/D/02/BCK/USA) | acid |  |
|  | ABX59502 (25/H/88/BCK/USA) |  |  |
| UL32 | EU104896 (12/D/02/BCK/USA) | Nucleotide | [6] |
|  | EU104897 (24/H/91/BCK/USA) |  |  |
|  | EU104898 (25/H/88/BCK/USA) |  |  |
|  | ABX59443 (12/D/02/BCK/USA) | Amino |  |
|  | ABX59444 (24/H/91/BCK/USA) | acid |  |
|  | ABX59445 (25/H/88/BCK/USA) |  |  |
| UL10 | EU104926 (25/H/88/BCK/USA) | Nucleotide | [6] |
| (glycoprotein M) | EU104937 (24/H/91/BCK/USA) |  |  |
|  | EU104938 (12/D/02/BCK/USA) |  |  |
|  | ABX59473 (25/H/88/BCK/USA) | Amino |  |
|  | ABX59484 (24/H/91/BCK/USA) | acid |  |
|  | ABX59485 (12/D/02/BCK/USA) |  |  |

* vac, vaccine; EU, Europe; USA, United States of America; Aus, Australia; Chi, China; Ita, Italy

References

1. Lee SW, Devlin JM, Markham JF, Noormohammadi AH, Browning GF, et al. (2011) Comparative analysis of the complete genome sequences of two Australian origin live attenuated vaccines of infectious laryngotracheitis virus. Vaccine 29: 9583-9587.

2. Lee SW, Markham PF, Markham JF, Petermann I, Noormohammadi AH, et al. (2011) First complete genome sequence of infectious laryngotracheitis virus. BMC Genomics 12: 197.

3. Chandra YG, Lee J, Kong BW (2012) Genome sequence comparison of two United States live attenuated vaccines of infectious laryngotracheitis virus (ILTV). Virus Genes 44: 470-474.

4. Spatz SJ, Volkening JD, Keeler CL, Kutish GF, Riblet SM, et al. (2012) Comparative full genome analysis of four infectious laryngotracheitis virus (Gallid herpesvirus-1) virulent isolates from the United States. Virus Genes 44: 273-285.

5. Poulsen DJ, Burton CR, O'Brian JJ, Rabin SJ, Keeler CL, Jr. (1991) Identification of the infectious laryngotracheitis virus glycoprotein gB gene by the polymerase chain reaction. Virus Genes 5: 335-347.

6. Oldoni I, Garcia M (2007) Characterization of infectious laryngotracheitis virus isolates from the US by polymerase chain reaction and restriction fragment length polymorphism of multiple genome regions. Avian Pathol 36: 167-176.

7. Oldoni I, Rodriguez-Avila A, Riblet S, Garcia M (2008) Characterization of infectious laryngotracheitis virus (ILTV) isolates from commercial poultry by polymerase chain reaction and restriction fragment length polymorphism (PCR-RFLP). Avian Dis 52: 59-63.

8. Waidner LA, Morgan RW, Anderson AS, Bernberg EL, Kamboj S, et al. (2009) MicroRNAs of Gallid and Meleagrid herpesviruses show generally conserved genomic locations and are virus-specific. Virology 388: 128-136.

9. Moreno A, Piccirillo A, Mondin A, Morandini E, Gavazzi L, et al. (2010) Epidemic of infectious laryngotracheitis in Italy: characterization of virus isolates by PCR-restriction fragment length polymorphism and sequence analysis. Avian Dis 54: 1172-1177.
